# Chemical recycling and repolymerization of poly(ethylene terephthalate), poly(ethylene furanoate), and their copolyesters

**DOI:** 10.1039/d6ra02625g

**Published:** 2026-07-06

**Authors:** Lauri Välinen, Hossein Baniasadi, Jukka Niskanen

**Affiliations:** a Polymer Synthesis Technology, School of Chemical Engineering, Aalto University Kemistintie 1 02150 Espoo Finland jukka.niskanen@aalto.fi

## Abstract

Replacing fossil-based plastics with bio-based ones is vital for future sustainability. However, bio-based plastics are not recycled within current recycling feeds, as polymers generally do not mix. Hence, mixing bio-based polymers with existing mechanical recycling feeds would yield materials of poor quality. For efficient recycling and as drop-in alternatives, bio-based polymers must have similar properties to the polymers in the feed they are mixed with. Chemical recycling and repolymerization can overcome incompatibility challenges by yielding copolymers. However, the question is: are the properties of the copolymers similar to those of the virgin homopolymers? Poly(ethylene furanoate) (PEF), a bio-based polymer, has similar properties to poly(ethylene terephthalate) (PET), such as high melting temperature, thermal stability, crystallinity, and gas-barrier properties. Both PEF and PET can be chemically recycled by solvolytic methods, and the obtained monomers can be repolymerized into copolymers that have similar properties to the virgin polymers. We have chemically recycled PET and PEF, repolymerized the recyclates into homo- and copolymers, and investigated the properties of the resulting polymers. We were able to repolymerize high-molecular weight polymers (*M*_w_ 63.5–123.4 kg mol^−1^) with good mechanical properties (up to 43.2 MPa). Collecting PEF and PET waste in the same recycling feed and chemically recycling them make the utilization of bio-based plastic feasible on a global scale, while utilizing existing recycling streams.

## Abstract 2

Glycolysis was used to chemically recycle PET bottles and PEF polymer into virgin-quality copolymers with sufficient mechanical properties.

## Graphical abstract

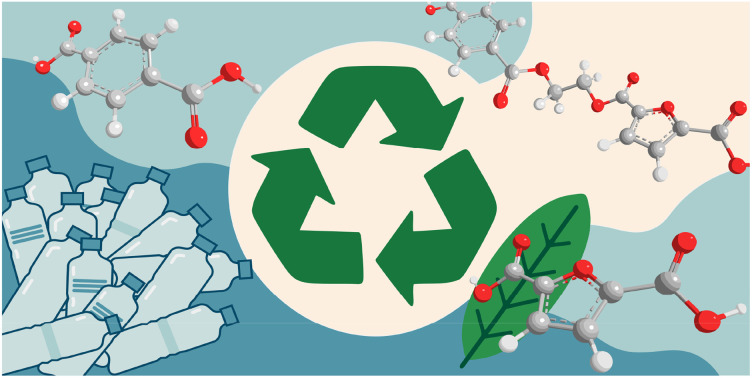

## 1. Introduction

Plastics are an integral part of modern life due to their remarkable properties, light weight, durability, processability and low cost. Annual global polymer production has reached 430 Mt.^1^ In 2020, 367 Mt of plastic was used, and consumption is predicted to rise to 874 Mt per year in 2050.^2^ Polyolefins account for roughly 58% of the annual production, while poly(ethylene terephthalate) (PET) represents about 6%. However, of the textile fibre production, almost 59% is polyester (PET).^3^ Only 9.5% of the polymers produced are recycled, while just 0.6% are bio-based polymers.^1^

The staggering and ever-increasing volume of produced plastics, which turn into plastic waste, and possibly microplastics makes plastics problematic to consumers. Hence, the positive aspects of plastics, *e.g.* in packaging, are sometimes overlooked. Replacing plastic packages with metal-, glass-, or wood-based materials could increase CO_2_ emissions by more than 35%.^4^ The European Union has implemented regulation stating that all plastic packaging should be recyclable by 2030, and that PET containers should contain at least 30% of recycled material by 2030 and 65% by 2040.^5^ Decreasing the use of non-renewable materials in packaging requires both better recycling technologies and packaging materials made from renewable resources. However, most renewable or bio-based materials, such as PLA,^6^ are not compatible with existing recycling streams, and they require separate processes for recycling or fermentation.^7^

Mechanical recycling is the preferred recycling method for plastics. In mechanical recycling, the plastics are sorted, ground, washed, melted, and extruded into pellets before reprocessing into new goods. The downside is that it often degrades the polymers and the recycled pellets may contain impurities and different grades of polymers, *i.e.* the market average of grades of the polymer and additives, and are therefore not suitable for *e.g.* food applications.^8,9^ Hence, mechanically recycled plastics are downgraded to products of lesser value. In addition, due to the degradation of the polymers, mechanically recycled polymers may release more microplastics than virgin polymers.^10^ PET is mechanically recycled from bottles to bottles or downgraded from bottles to textile fibres.^11^ However, mechanical recycling is currently the most viable recycling method due to low costs and emissions.

In chemical recycling, polymers are broken down into their monomers, or other raw materials,^12,13^ either by solvolytic methods or pyrolysis. Solvolytic methods include hydrolysis, aminolysis, methanolysis, and glycolysis and are suitable for chemical recycling of polyesters,^13–15^ polyamides^16–18^ and polyurethanes.^19–21^ These polymers all have bonds that can be hydrolysed.

Metal catalysts are widely used in the chemical recycling of plastics. Ir, Pt, Re, Ru, Sr, and Ti are used in the pyrolysis (>500 °C) of poly(ethylene) and poly(propylene) into gases, liquid hydrocarbons, and waxes.^22^ Metal (*e.g.* Co, Ir, Mo, and Ru) salts and oxides are used to catalyse the depolymerization of polycarbonates, polyamides, and polyesters.^23^ Metal (Co, Mn, Pb, Zn) acetates and (K, Na) carbonates are efficient transesterification catalysts and therefore suitable in solvolytic depolymerization of PET. Of the acetates, zinc acetate has the highest activity, while of the carbonates, K_2_CO_3_ has the highest activity. However, both types of catalysts require rather high reaction temperatures (>190 °C) and rather long reaction times (6–10 h). The high temperatures and the long reaction times are related not only to catalytic activity but also to reaction conditions; *i.e.*, if the reaction occurs under heterogeneous conditions of PET flakes, in *e.g.*, ethylene glycol or if the PET is dissolved (homogeneous conditions).^24–26^

Glycolysis utilizes ethylene glycol to break down polyesters into oligomers and low-molecular weight products, *e.g.* bis(2-hydroxyethyl) terephthalate (BHET) in the case of PET.^15,27,28^ BHET, an intermediate product in virgin PET synthesis, melts at a low temperature (106 °C) and can be polymerized into PET. Pyrolysis, where plastics are broken down under high heat, is suitable for polyolefins and mixed plastic waste, and is currently being developed on an industrial scale.^29^ However, it is very energy-demanding and the yields and capability to yield desired products are still under intense development.^11^ Chemical recycling is an advantageous and attractive method, as it yields polymers with properties identical to virgin polymers and downgrading of the plastics is avoided. Chemical recycling can be used to produce copolymers with a wide range of properties, depending on the materials used.^30–33^ However, copolymerization can make the resulting polymers difficult, or even impossible to recycle.

Poly(ethylene furanoate) (PEF) is a polyester with very similar properties to PET and has gained a lot of attention in recent years.^34–40^ PEF is seen as a viable alternative to PET, as it is bio-based and has better gas-barrier properties.^41,42^ Both polymers contain ethylene glycol as a monomer, which is why chemical recycling by glycolysis is a feasible option for both PET and PEF. By combining the recycling feeds of PET and PEF, it is possible to bring drop-in bio-based alternatives to market, which can be produced, processed, turned into products, and recycled using existing methods. PEF is compatible with PET up to 5% in mechanical recycling,^43^ and by chemical recycling, compatibility could be further increased.

In this paper, we chemically recycle PET and PEF by glycolysis into oligomers and monomers, which are then repolymerized into homo- and copolymers, in order to investigate if the PEF content in the recycled polymers can be increased above 5%, without compromising the properties of the polymers.

While the glycolysis of PET has been extensively studied,^15,27,28,44–52^ the glycolysis and repolymerization of PEF are less so.^53–59^ For convenient purification, we targeted oligomers rather than BHET (for PET) or BHEF (for PEF).

The molecular weights of the obtained polymers are comparable to those of virgin PET (*M*_w_ 63.5–123.4 kg mol^−1^). The mechanical properties indicate that PEF could be a drop-in solution to replace PET, and the resulting poly(ethylene terephthalate-*co*-ethylene furanoate) (PETF) copolymers could aid in a gradual transition to bio-based alternatives.^60^ A series of copolymers with various ratios of terephthalic acid (TPA) to FDCA obtained from PEF and PET were prepared to understand the structure–property relationships of the copolymers.

The differences in the crystallization behaviour between PET and PEF limit the composition of the copolymers.^60^ While both polymers are semi-crystalline, we observed that the time required for crystallization increased with the 2,5-furandicarboxylic acid (FDCA) content in the copolymers. This could lead to challenges in industrial processes where rapid crystallization is an important factor. However, challenges with crystallization could be avoided by adjusting the ratio of TPA and FDCA. On the other hand, the slower crystallization behaviour may be beneficial in applications where crystallinity is induced by mechanical means, for example, blow moulding and fibre drawing.

## 2. Experimental

### 2.1 Materials

2,5-Furandicarboxylic acid 98% was obtained from AA Blocks, USA. Ethylene glycol (ReagentPlus ≥99%), ethylene glycol (EMPLURA 99%) and titanium(iv) isopropoxide (97%) were ordered from Sigma-Aldrich. Zinc acetate (>98.0%), pentaerythritol tetrakis[3-(3,5-di-*tert*-butyl-4-hydroxyphenyl)propionate] (PTP >95%) and sodium trifluoroacetate (>98%) were purchased from TCI. 1,1,1,3,3,3-Hexafluoro-2-propanol (99%) was ordered from Apollo Scientific and was recycled by distillation for size exclusion chromatography. Trifluoroacetic acid-D (99.5% D) was ordered from Thermo Fisher Scientific. Chloroform-D (99.8% D) was obtained from Eurisotop. All materials were used as received. The water used was purified by reverse osmosis (type 3). PET bottles used in the study were regular soda bottles obtained from a local supermarket.

### 2.2 Polyester synthesis

**Scheme 1:**
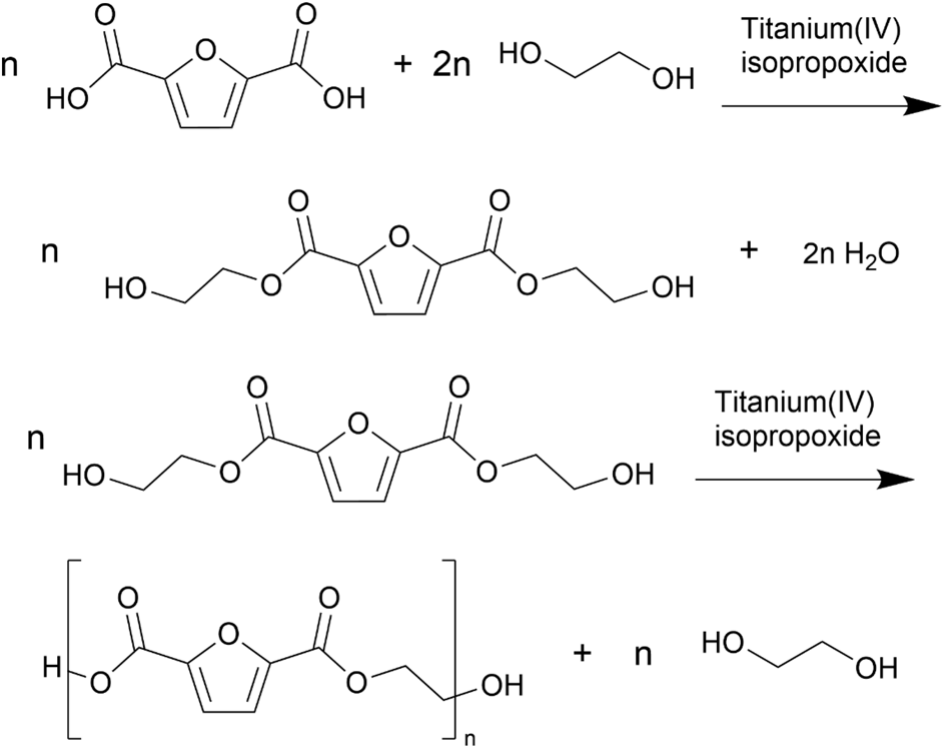
The synthesis of poly(ethylene furanoate).

Poly(ethylene furanoate) (PEF) was polymerized in a two-step synthesis, starting with an esterification step and followed by a polycondensation step (Scheme 1). The polymerizations were conducted in a 100 mL Büchi Novoclave stirred autoclave reactor using titanium(iv) isopropoxide (TTIP) as a catalyst and pentaerythritol tetrakis[3-(3,5-di-*tert*-butyl-4-hydroxyphenyl)propionate] (PTP) as an antioxidant. The ratio between the diacid (FDCA) and diol was targeted to be around 1 : 3. The reaction temperature was monitored and controlled from inside the reactor. The pressure in the reactor was reduced using an IKA VC 10 pro vacuum controller and a Vacuubrand RZ 2.5 rotary vane pump reaching <1 mbar. The reactor was equipped with a propeller stirrer, and a stirring speed of 100 rpm was used for polyester synthesis. In a typical polymerization, 2,5-furandicarboxylic acid (FDCA) (29.93 g, 0.19 mol), ethylene glycol (EG) (33.33 g, 0.54 mol, ≥99%), and antioxidant (0.32 g, 0.27 mmol) were loaded in the reactor and stirred into a slurry. TTIP (0.10 g, 0.37 mmol) was added to the slurry. The reactor was purged with nitrogen for 20 minutes, after which the nitrogen purge was decreased to prevent sublimation of FDCA, and the temperature was set to 180 °C. As the reaction mixture heated up to 180 °C, the slurry turned into a solution and eventually into a melt as the reaction proceeds. After 1 hour, the temperature was increased to 230 °C. Once the reactor reached 230 °C, the pressure was gradually decreased for 20 minutes, until the pressure was <1 mbar. During the polymerization, the torque of the mechanical stirrer was observed to increase from 4 Ncm to 12 Ncm due to increased viscosity. After 17 hours, the reactor was brought to atmospheric pressure with nitrogen, and the polymer melt was collected from the reactor under nitrogen flow. ^1^H-NMR (400 MHz, TFA-d + CDCl_3_ (4 : 6), *δ* ppm): 4.07 (–CH_2_–), 4.15 (–CH_2_–), 4.60 (–CH_2_–), 4.65 (–CH_2_–), 4.75 (–CH_2_–), and 7.35 (=CH–) (Fig. 2b).

PEF was also prepared in a glass flask. In a typical reaction, 103.8 g (0.6650 mol) of FDCA, 106.8 g (1.721 mol) of EG, 1.105 g (0.9383 mmol) of antioxidant and 0.192 g (0.676 mmol) of TTIP were loaded into a 2-neck 250 mL glass flask, equipped with a magnetic stir bar, a nitrogen inlet, and a distillation column. The stirrer was set to 500 rpm and the temperature to 180 °C, and the system was purged with N_2_. The flask was kept at 180 °C for 2.5 hours while water was distilled off. Then the distillate was emptied, the temperature was increased to 230 °C, the nitrogen flow was removed, and the pressure was reduced to 10 mbar using an IKA VACSTAR digital vacuum pump with a VC 10 lite vacuum controller. After 20 hours at 10 mbar, the flask was pressurized with nitrogen, and the polymer melt was collected. ^1^H-NMR (400 MHz, TFA-d + CDCl_3_ (4 : 6), *δ* ppm): 4.07 (–CH_2_–), 4.15 (–CH_2_–), 4.60 (–CH_2_–), 4.65 (–CH_2_–), 4.75 (–CH_2_–), and 7.35 (=CH–). The ^1^H-NMR spectrum is presented as SI (Fig. S3).

### 2.3 Glycolysis

**Scheme 2:**
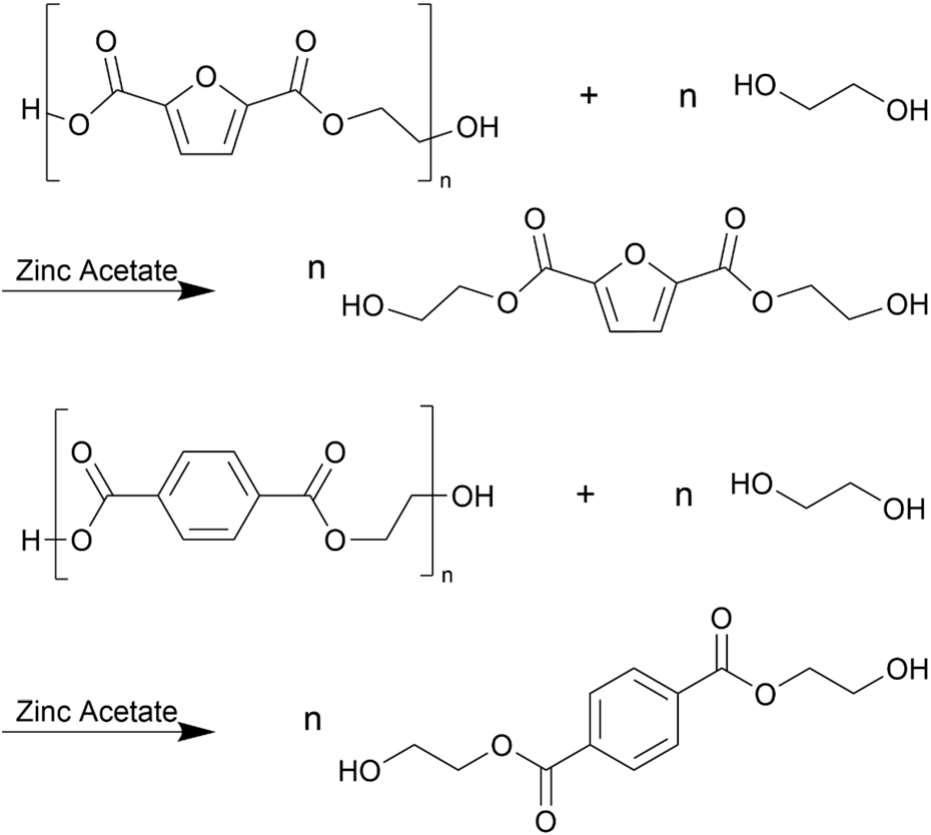
The glycolysis of poly(ethylene furanoate) (PEF) into bis(2-hydroxyethyl)furan-2,5-dicarboxylate (BHEF) and poly(ethylene terephthalate) (PET) into bis(2-hydroxyethyl)terephthalate (BHET), using zinc acetate as catalyst.

The glycolysis reactions were conducted in a glass flask using mechanical stirring (50 rpm) (Scheme 2). For the glycolysis of PET bottles, the labels and bottle caps were removed, and the glue residue from the labels was washed off with soap and water. The bottles were shredded using a Retsch SM300 cutting mill equipped with an 8 mm sieve and a cyclonic separator to remove dust from the PET flakes. An EG : PET ratio of 1 : 1 to 2.5 : 1 was used in the reactions. In a typical glycolysis reaction of PET, 20.0 g of PET flakes were mixed with 50.0 g (0.8 mol) EG and 0.09 g (1.0 mmol) zinc acetate in a 500 mL flask. The flask was purged with nitrogen and heated to 180 °C under nitrogen flow. The mixture was cooled to room temperature after 24 hours and poured into 200 mL of water. The resulting slurry was left to separate overnight at 4 °C. The BHET was filtered, washed with 600 mL of water, and dried *in vacuo* at 60 °C for 72 hours. 18.5 g of BHET was obtained (yield 70.0%). ^1^H-NMR (400 MHz, CDCl_3_ (4 : 6), *δ* ppm): 4.09 (–CH_2_–), 4.63 (–CH_2_–), 4.75 (–CH_2_–), and 8.15 (=CH–) (Fig. 2c).

Similarly, for the glycolysis of PEF, 20.0 g of crushed PEF and 20.0 g (0.3 mol) of EG were charged into a 250 mL flask together with 0.12 g (0.65 mmol) of zinc acetate. The flask was flushed with nitrogen and heated to 180 °C. After 21 hours, the homogeneous yellow liquid was poured into 200 mL of cold water. The resulting slurry was kept at 4 °C for 72 hours. The precipitated solids were filtered, washed with cold water, and dried *in vacuo* at 60 °C. 7.5 g of BHEF was obtained (yield 28.0%). ^1^H-NMR (400 MHz, TFA-d : CDCl_3_ (4 : 6), *δ* ppm): 4.09 (–CH_2_–), 4.63 (–CH_2_–), 4.75 (–CH_2_–), and 7.35 (=CH–) (Fig. 2a).

### 2.4 Repolymerization

The repolymerizations of the oligomers into homopolymers (rPEF and rPET) and copolymers (poly(ethylene terephthalate-*co*-ethylene furanoate), rPETF) were made in a Büchi Novoclave reactor in 60 g batches, using titanium(iv) isopropoxide (TTIP) as a catalyst. The polycondensation reactions of rPEF were conducted at 230 °C, the rPETF copolymers at 250 °C and rPET at 270 °C.

A typical copolymerization (rPETF 25–75) was conducted as follows: BHET (15.4 g, 0.06 mol) and BHEF (45.9 g, 0.19 mol) were loaded into the reactor and ground together, followed by the addition of TTIP (0.20 g, 0.70 mmol) and antioxidant (0.30 g, 0.25 mmol). The reactor was heated to 120 °C under nitrogen flow to melt the monomers. After the monomers had melted, the reactor was closed, the nitrogen flow was decreased, and the temperature was increased to 230 °C. After the reactor reached the desired temperature of 230 °C, the nitrogen flow was stopped, and the pressure was reduced to <1 mbar. After one hour, the reaction temperature was further increased to the final temperature of 250 °C. After 17 hours, the reactor was repressurized, opened under nitrogen flow, and the polymer melt was collected from the reactor. The torque of the stirrer was observed to increase from 4 Ncm to 15–92 Ncm during the first 10–15 hours, as the viscosity of the polymer melt increased during the polymerization. Polymers containing more terephthalic acid were observed to have higher viscosities in comparison to the other copolymers, most likely due to the higher melting temperature of PET. Details of the repolymerizations are presented in Table 1. In case of rPET, reaction was stopped after 4 hours.

**Table 1:** Details of the repolymerizations of bis(2-hydroxyethyl)furan-2,5-dicarboxylate (BHEF) and bis(2-hydroxyethyl)terephthalate (BHET) into homo- and copolyesters poly(ethylene terephthalate) (rPET), poly(ethylene furanoate) (rPEF) and poly(ethylene terephthalate furanoate) (rPETF). Titanium(iv) isopropoxide (TTIP) was used as a catalyst and pentaerythritol tetrakis[3-(3,5-di-*tert*-butyl-4-hydroxyphenyl)propionate] as an antioxidant

Sample name	BHET		BHEF		TTIP		Antioxidant	
	g	mol	g	mol	g	mmol	g	mmol
rPET	60.02	0.24	—	—	0.20	0.71	0.30	0.25
rPETF 75–25	45.01	0.18	15.00	0.061	0.21	0.75	0.30	0.25
rPETF 50–50	31.75	0.12	31.88	0.13	0.18	0.64	0.31	0.26
rPETF 25–75	15.42	0.061	45.90	0.19	0.20	0.70	0.30	0.25
rPEF	—	—	59.95	0.25	0.20	0.70	0.31	0.26

### 2.5 Characterization methods

#### 2.5.1 Fourier-transform infrared spectroscopy

FTIR analysis was done with Bruker Alpha II ATR-FTIR spectrometer. A total of 128 scans were performed from 4000 cm^−1^ to 400 cm^−1^ with a resolution of 4 cm^−1^. The spectra were baseline-corrected and normalized using OPUS software. Data were plotted using OriginPro 2025.

#### 2.5.2 Nuclear magnetic resonance spectroscopy

The ^1^H-NMR spectra were recorded on a Bruker Avance NEO 400 MHz NMR spectrometer. 10 mg of sample was dissolved in a mixture of 0.4 mL deuterated trifluoroacetic acid and 0.6 mL of deuterated chloroform. 128 scans were collected with a relaxation delay of 5 s. The chloroform signal at *δ* = 7.26 ppm was used as a reference. The spectra were baseline-corrected and analysed using Bruker Topspin.

The diethylene glycol (DEG) content in the polymers was calculated using eqn (1):1
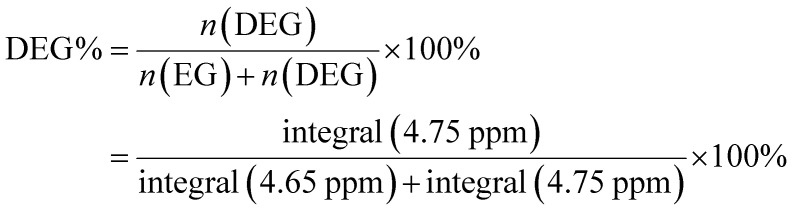


#### 2.5.3 Size exclusion chromatography

The number-average molecular weight (*M*_n_), the weight-average molecular weight (*M*_w_) and the dispersity (*Đ*) were determined using the Waters Arc HPLC system consisting of Waters Arc HPLC quaternary solvent manager-R, Waters Arc HPLC sample manager FTN-R, two Agilent HFIPgel 250 × 4.6 mm columns and an HFIPgel Guard 50 × 4.6 mm inside a Shimadzu CTO-40 C column oven at 40 °C. Wyatt Optilab refractive index detector was used for conventional calibration and as a concentration detector, and Wyatt DAWN multi-angle light scattering detector (MALS) was used for absolute molecular mass determination. 1,1,1,3,3,3-Hexafluoro-2-propanol (HFIP) with 0.02 M sodium trifluoroacetate was used as an eluent. The flow rate was set at 0.3 mL min^−1^. Samples at 2 mg mL^−1^ were prepared, and the injection volume was 50 µL. The instrument was calibrated with a set of narrow polymethyl methacrylate (PMMA) standards (Shodex) ranging from 2880 g mol^−1^ to 211 000 g mol^−1^ and was fitted using a first-order fit with R2 of 0.9917. Refractive index increment (d*n*/d*c*) values of 0.227 mL g^−1^ for PEF^61^ and 0.249 mL g^−1^ for PET^61^ were used with MALS. For copolymers the refractive index increment was estimated by mass fractions in copolymers according to eqn (2), where *W*_A_ is the weight fraction of polymer A in copolymer.^62^2
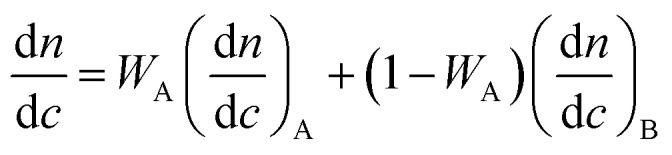


Light scattering detectors were normalized using a PMMA standard with *M*_p_ of 17 800 g mol^−1^ and *Đ* of 1.04, while band broadening and alignment were performed using a PMMA standard with *M*_p_ of 211 000 g mol^−1^ and *Đ* of 1.02.

#### 2.5.4 Differential scanning calorimetry

A TA Instruments Discovery DSC 250 differential scanning calorimeter was used to determine the glass transition temperature (*T*_g_), melting temperature (*T*_m_), crystallinity, and the crystallization rate of the polymers. *T*_g_ values are reported at the transition midpoint, and onset values are reported for *T*_m_. 5–10 mg samples were sealed in Tzero aluminium pans. The measurements were conducted under 50 mL min^−1^ nitrogen flow. The samples were heated to 280 °C at a rate of 50 °C min^−1^ to erase thermal history. The samples were then cooled to 170 °C and held isothermally at 170 °C for 4 hours to allow for the samples to crystallize. The samples were then cooled to 0 °C and heated at 10 °C min^−1^ to 280 °C to record the glass transition, melting temperature, and enthalpy of melting.

#### 2.5.5 Mechanical testing

The films for tensile strength and dynamic mechanical analysis were prepared by hot pressing at 250 °C using polyimide sheets as a frame. The films were cut into shapes according to ASTM D638 Type V standards (dog bones with 3.18 mm width) and characterized using Instron 4204 universal testing instrument equipped with a 5 kN load cell. Samples were stretched at a rate of 5 mm s^−1^, and three repetitions were made with each sample.

#### 2.5.6 Thermogravimetric analysis

A TA Instruments TGA5500 was used to study the thermal decomposition of polyesters and monomers. Samples of 8–10 mg were loaded into 100 µL platinum pans and heated at 10 °C min^−1^ from 30 to 800 °C under nitrogen flow. The degradation temperature (*T*_d_) was determined at 5% mass loss.

## 3. Results and discussion

To mimic the actual chemical recycling processes of both PEF and PET, virgin materials were subjected to glycolysis under similar conditions. Virgin PEF was obtained by synthesizing it on site, and virgin PET was obtained from commercial bottles. Both were then subjected to glycolysis to obtain monomers, which were later used in repolymerizations. A series of copolymers with varying contents of the glycolysis products were prepared to obtain copolymers with 75/25, 50/50, and 25/75 wt% of terephthalic acid (TPA) to furan dicarboxylic acid (FDCA) oligomers. In addition, homopolymers were also made. To understand the effect of monomer composition on the thermal and mechanical properties of the polymers, and thus the feasibility of chemically recycling these two polymers in the same feed, the obtained polymers were characterized by DSC, TGA, and tensile testing.

### 3.1 Virgin polymers and glycolysis

**Fig. 1:**
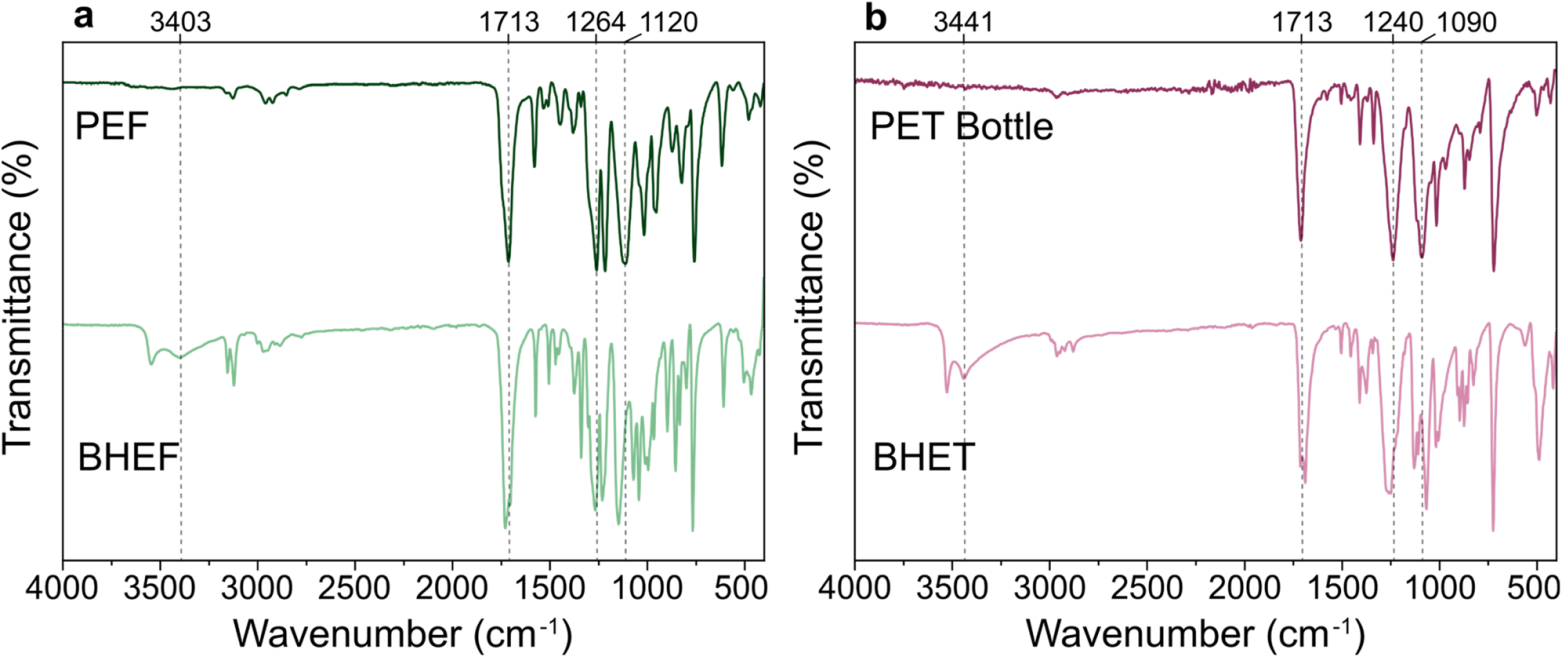
(a) FTIR spectra of virgin PEF and BHEF (b) FTIR spectra of virgin PET and BHET.

**Fig. 2:**
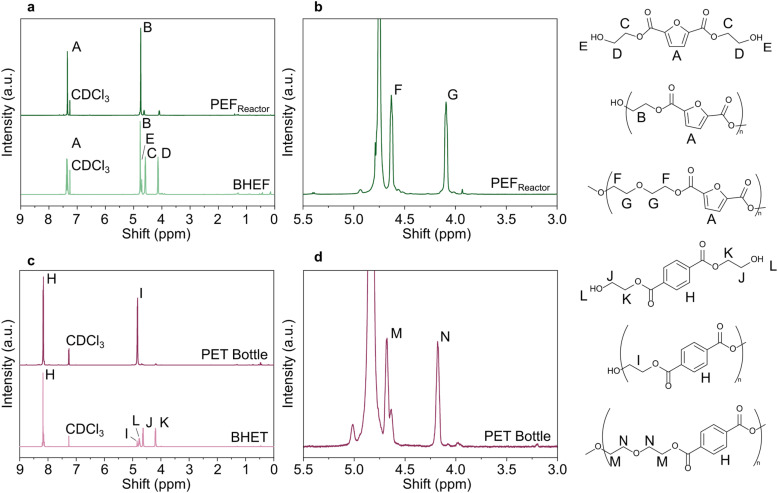
(a) ^1^H-NMR spectra of virgin PEF and BHEF (b) ^1^H-NMR spectra of PEF ranging from 5.5–3 ppm (c) ^1^H-NMR spectra of virgin PET and BHET (d) ^1^H-NMR spectra of virgin PET ranging from 5.5–3 ppm.

Virgin PEF was obtained from two polymerizations; one batch was prepared in a stainless-steel reactor and one batch in a glass flask. The stainless-steel reactor was equipped with a mechanical stirrer and a more powerful vacuum pump than the one that was used with the glass flask (<1 mbar compared to 10 mbar). This change had an effect on the molecular weight of the obtained polymers, as the PEF obtained from the reactor had a higher molecular weight (*M*_w_ 50.9 kg mol^−1^, *Đ* 3.14) than the one obtained from the glass flask (*M*_w_ 22.6 kg mol^−1^, *Đ* 1.69). The obtained polymers had molecular weights in the order of what has been reported earlier (42.2–98.3 kg mol^−1^).^34^ The mechanical stirring and lower pressure in the reactor both contributed to improved removal of released ethylene glycol from the polymer melt during the polymerization. A titanium-based catalyst was chosen, as they are known to be efficient catalysts for both the esterification step and the polycondensation step during the polymerization process.^63^ Zinc acetate, which was used in the depolymerization, can be used as a catalyst for the esterification step, however, a cocatalyst is needed for the polycondensation step.^63,64^ The FTIR spectra are presented in Fig. 1 and the ^1^H-NMR spectra are presented in Fig. 2 and in SI.

The glycolysis reactions of both PEF (*M*_w_ 22.6 kg mol^−1^, *Đ* 1.69) and PET (*M*_w_ 55.8 kg mol^−1^, *Đ* 1.50) were conducted at 180 °C overnight with an excess of ethylene glycol using a zinc acetate catalyst, and the obtained oligomers would later be used in the polymerization of copolymers.^15,46,65,66^

The virgin polymers (PEF and PET) and glycolysis products (BHEF and BHET) were characterized by FTIR and ^1^H-NMR. The characteristic carbonyl absorbance band at 1713 cm^−1^ and the ester bond at 1240 and 1090 cm^−1^ are observed in the FTIR spectra of PET. In the case of PEF, the bands of the ester bond are slightly shifted to 1264 and 1120 cm^−1^ (Fig. 1a and b). The furan ring has distinctive absorbance bands at 757, 827, 874, 1020, and 1580 cm^−1^, which can be observed for both PEF and BHEF. The aromatic bands from terephthalic acid in PET and BHET are observed at 724, 870, and 1015 cm^−1^. The bands from the hydroxyl groups of BHEF and BHET can be observed at 3403 and at 3441 cm^−1^.

In the ^1^H-NMR spectra, the aromatic signals from the furan ring and the benzene ring are observed at 7.35 and 8.15 ppm, respectively (Fig. 2a and c). The methylene signals from ethylene glycol are observed at 4.75–4.81 ppm. The residual signals from the ethylene glycol end groups are observed at 4.15 and 4.6 ppm, however, as the trifluoroacetic acid forms esters with the hydroxyl end groups, these signals cannot be used for quantitative analysis.^67^ Diethylene glycol (DEG) moieties are observed at 4.10 and at 4.65 ppm (Fig. 2b and d). The formation of diethylene glycol is a known issue with PET and PEF, and it is known to affect both the mechanical and thermal properties of the polymers, causing a decrease in the glass transition temperature (*T*_g_) and melting temperature (*T*_m_).^34,68–73^ For PET, the *T*_m_ is reported at 250–265 °C, and a decrease in the *T*_m_ from 270 to 250 °C occurs as DEG content increases from 0.6% to 3.64%.^74^ A high amount of ethylene glycol in the synthesis increases the formation of diethylene glycol groups, which explains the over 10% DEG content in virgin PEF. Many catalysts, including TTIP, are known to also catalyse the formation of DEG groups.^69,72,73^ The DEG content (3–11%) in the polymers was determined from the signals at 4.1 and 4.8–4.75 ppm from diethylene glycol and ethylene glycol moieties, respectively, and the values are presented in Table 2. The DEG content in the synthesized polymers is higher than what is observed for virgin PET (3%). Hence in future studies we aim to change the catalyst and reduce the excess of EG to reduce the amount of DEG in the obtained polymers. No signals from DEG moieties can be observed in the spectra of the glycolysis products BHEF and BHET. End group analysis of the chemically recycled products shows that the average degree of polymerization for BHEF/PEF is 3.0 and for BHET/PET 1.4. It should be noted that although the glycolysis of PEF yielded slightly longer oligomers, the purification of the BHET monomer was observed to be more facile, as it precipitated more readily from water than the BHEF/PEF oligomers. In addition, signals from both the antioxidant and the catalyst can be observed in the NMR spectra. The signals from the antioxidant are observed at 1.42 ppm (–CH_3_), 2.77 and 2.91 ppm (–CH_2_–), and 3.94 ppm (–OH), and the aromatic ring at 7.00 and 7.02 ppm. The quintet from TTIP is observed around 4.27 ppm and the methyl doublet at 1.15–1.11 ppm. The full ^1^H-NMR spectra of the polymers and glycolysis products are presented in the SI (Fig. S1–S5).

**Table 2:** Summary of ^1^H-NMR data analysis and molecular weight data of virgin poly(ethylene terephthalate) (PET) and poly(ethylene furanoate) (PEF); and re-poly(ethylene furanoate) (rPEF) and re-poly(ethylene terephthalate-*co*-ethylene furanoate) (rPETF) copolymers obtained from chemically recycled PEF and PET

Sample	^1^H-NMR			SEC (MALS)			
	FDCA (%)	TPA (%)	DEG/EG (%)	*M*_n_ (g mol^−1^)	*M*_w_ (g mol^−1^)	*Đ*	d*n*/d*c* value
PET bottle	—	100.0	2.6	37 300	55 800	1.50	0.249
PEF_Flask_	100	—	4.7	13 400	22 600	1.69	0.227
PEF_Reactor_	100	—	10.7	16 200	50 900	3.14	0.227
rPEF	100	—	6.7	28 300	63 500	2.24	0.227
rPETF 25–75	77	23	5.0	40 300	109 900	2.73	0.233
rPETF 50–50	48	52	9.1	43 600	123 400	2.83	0.238
rPETF 75–25	23	77	6.2	36 000	94 100	2.62	0.244
rPET	0	100	6.7	42 800	113 000	2.64	0.249

### 3.2 Repolymerization

**Fig. 3:**
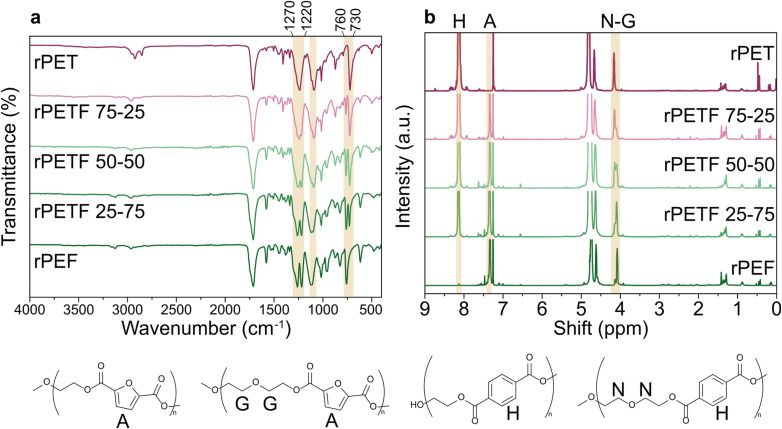
(a) FTIR spectra (b) ^1^H-NMR spectra of re-poly(ethylene furanoate) (rPEF) and re-poly(ethylene terephthalate-*co*-ethylene furanoate) (rPETF) copolymers prepared from chemically recycled poly(ethylene terephthalate) and poly(ethylene furanoate).

**Fig. 4:**
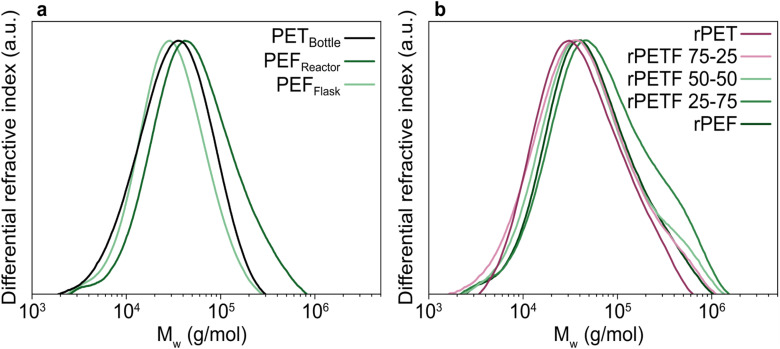
Size exclusion chromatography (SEC) eluograms of (a) virgin poly(ethylene terephthalate) (PET) and poly(ethylene furanoate) (PEF); and (b) re-poly(ethylene furanoate) (rPEF) and re-poly(ethylene terephthalate-*co*-ethylene furanoate) (rPETF) copolymers obtained from chemically recycled PEF and PET.

A series of copolymers, including two homopolymers, was prepared from the glycolysis products, with varying ratios of FDCA to TPA, 100/0, 75/25, 50/50, 25/75, and 0/100 wt%, in a stainless-steel reactor. The molecular weights and the monomer ratios of all polymers are shown in Table 2. The monomer ratios of FDCA and TPA, as well as ethylene glycol (EG) and diethylene glycol (DEG) were determined from ^1^H-NMR spectra (Fig. 3b and S6–S10). Size exclusion chromatography equipped with a multi-angle light scattering detector was used to determine the absolute weight-average molecular weight (*M*_w_), number-average molecular weight (*M*_n_) and dispersity (*Đ*) of the polymer samples (Fig. 4a and b). In addition, the molecular weights of the polymers were estimated with PMMA standards for comparison (SI, Table S1).

The FTIR spectra of the repolymerized copolymers are similar to the virgin polymers: the carbonyl absorbance band is observed at 1713 cm^−1^ for all polymers as expected. In the copolymers, bands from both FDCA and TPA are observed at 730 and 760 cm^−1^, respectively. The relative intensities are observed to change according to the ratio of FDCA to TPA, as the band for terephthalate (730 cm^−1^) decreases and the band from the furan ring increases (760 cm^−1^) with increasing FDCA content in the copolymers. A similar observation can be made for the bands from the ester bond at 1090 to 1120 cm^−1^ and the bands at 1220 cm^−1^ and 1580 cm^−1^ from the furan ring, which can be seen to increase with increasing FDCA content. Hence, the presence of both FDCA and TPA in the copolymers can be confirmed by FTIR.

Similar trends can be observed in the ^1^H-NMR spectra. The intensities of the signals originating from the furan ring (7.35 ppm) and the benzene ring (8.15 ppm) change with the copolymer composition. The copolymer compositions, *i.e.* the ratio of FDCA to TPA, could be determined from these signals, and were determined to be within 2% of the theoretical compositions (Table 2), similar to what has been reported by Konstantopoulou *et al.*^60^ The methylene signal from ethylene glycol is observed at 4.8 ppm. However, the signals originating from DEG, the protons closer to the ether group, are separated into two signals at 4.10 and 4.16 ppm, and the signals from these methylene groups are observed to move downfield from 4.10 ppm to 4.16 ppm with increasing TPA content in the copolymers. Similar observations have been made by Konstantopoulou *et al.*^60^ The signal from the hydroxyl end groups can be observed at 4.13 ppm in rPEF,^34,75^ however, this signal overlaps with the signal from virgin polymers.

The DEG content is similar in the repolymerized polymers (5–9%) to the virgin polymers (3–11%, see Table 2). In addition to the amount of ethylene glycol present and the catalyst used, the polymerization temperature is known to influence the formation of DEG, as higher polymerization temperatures are known to increase the formation rate of DEG. In the case of PET, increasing the temperature from 270 °C to 290 °C can more than double the formation rate of diethylene glycol groups.^71^

The molecular weights of the polymers are presented in Table 2, and the SEC eluograms in Fig. 4. Absolute *M*_w_ was determined using multi-angle light scattering (MALS). The refractive index increments for the copolymers were obtained by using mass fractions in the copolymers.^62^ Calibration using PMMA standards overestimates the molecular weights of PEF samples, as can be observed from the molecular weight data, presented in the SI (Table S1). For example, for PEF_Reactor_, PMMA calibration gives *M*_w_ of 91.7 kg mol^−1^, whereas the *M*_w_ determined by MALS is 50.9 kg mol^−1^. This overestimation is due to different elution times in the columns due to the differences in hydrodynamic radii. Hence, MALS provides the means to compare the actual molecular weights of the synthesized polymers and copolymers.

In the polymerizations, we aimed for a similar molecular weight to that of the PET from the commercial bottles (*M*_w_ 55.8 kg mol^−1^, *Đ* 1.5) in our syntheses. Stirring efficiency, pressure, and temperature were observed to affect the molecular weight of the polymers, as one could expect. PEF synthesized in a flask (*M*_w_ 22.6 kg mol^−1^, *Đ* 1.69) had a lower *M*_w_ than the one prepared in the stainless-steel reactor (*M*_w_ 50.9 kg mol^−1^, *Đ* 3.14). The difference can be attributed to improved stirring (magnet *vs.* mechanical stirring) and lower pressure during the polycondensation phase of the polymerization (10 mbar in the flask and <1 mbar in the reactor). It should be noted that the polycondensation step was three hours longer in the glass flask than in the reactor, and by further increasing the time, similar molecular weights should be obtained. However, a longer polycondensation step also increases the side reactions (formation of acetaldehyde, carboxyl end groups, and vinyl end groups) at this high temperature.^63^ The polymerization yielded polyesters with slightly higher dispersities (*Đ* 2.24–2.83) than would be expected. Typical dispersities for polycondensation polymers are reported to be around 2.^64^ The copolymers have higher molecular weights (*M*_w_ 94.1–123.4 kg mol^−1^) than the PEF homopolymer (*M*_w_ 63.5 kg mol^−1^). This increase in *M*_w_ is due to higher conversions reached, when the reaction temperature is increased from 230 to 250 °C. The increase in the temperature lowers the viscosity of the polymer melts, which improves the removal of EG from the melt, yielding higher conversions. As PET is typically synthesized at 270–290 °C,^63^ 250 °C was chosen as the polymerization temperature for the copolymers, as we were concerned that 230 °C would not be sufficient. Looking at the molecular weights and dispersities of the polymers, the increase in temperature was suitable for PETF 75–25 and might have been unnecessary for PETF 25–75 and PETF 50–50, as the *M*_w_ of these polymers is significantly higher than that of the other copolymers (*M*_w_ 109.9 and 123.4 kg mol^−1^). The dispersity was also higher for the latter polymers. Obtaining high *M*_w_ polymers with low dispersity is challenging due to transesterification, where chains react together to form shorter, longer, and cyclic polymers, increasing dispersity.^64^ Titanium-based catalysts are known to also catalyse ester interchange reactions.^63^ Factors in reaction conditions that can increase dispersity are poor mixing caused by high viscosity and degradation. In addition, very viscous polymers tend to get stuck to the reactor walls and stirrer, slowing the diffusion of condensation products. In addition, a decrease in DEG content in the polymers is known to affect the *T*_g_ of the polymers,^68^ therefore the *T*_g_ of rPETF 50–50 is slightly lower than that of the other copolymers, as its DEG content is slightly higher (9.1%) than for the other copolymers (5.0 and 6.2%). Melting temperatures (*T*_m_) were observed for PEF homopolymers (188 and 192 °C), the copolymer with the highest amount of TPA, rPETF 75–25 (186 °C), and rPET at 225 °C. The slightly lower melting temperature of rPET is likely due to the increased amount of DEG from 2.6 to 6.7%. No *T*_m_ could be observed for the other two copolymers (SI Fig. S11). Konstantopoulou *et al.* have shown that PETF copolymers are amorphous and do not show any crystallization unless the comonomer content is small.^60^ The low or non-existent crystallinity of PETF copolymers is due to the random structure of the copolymers, which hinders the formation of crystallites. Two peaks for the melting temperature can be observed for PEF homopolymers and rPETF 75–25. Similar broad and multiple melting peaks were also observed by Konstantopoulou *et al.* and they are caused by cocrystallization^60^ and polymorphism.^76^ The degree of crystallinity (*X*_c_) of the polymers was calculated based on the reported values of 100% crystalline PET (140 J g^−1^),^77^ and PEF (137 J g^−1^),^78^ see Table 3. The degree of crystallinity of rPEF (35.5%) is in the range of what has been previously reported for PEF (13–39%) with similar molecular weights (*M*_w_ 35–50 kg mol^−1^).^53,60^ The degree of crystallinity of PEF_Reactor_ is significantly lower (5.5%) than that of rPEF, which is explained by the higher DEG content in the polymers; 10.7 *vs.* 4.7%, respectively. The degree of crystallinity of rPET is 23.9%, similar to what is observed for PET_Bottle_ (23.1%). Hence it can be concluded that the crystallinity of the polymers is significantly reduced by the copolymerization (homopolymers *vs.* copolymers) but is also affected by the DEG content (rPEF *vs.* REF_Reactor_, Tables 2 and 3).

**Table 3:** Thermal characterization and tensile testing data of poly(ethylene furanoate) (PEF); and re-poly(ethylene furanoate) (rPEF) and re-poly(ethylene terephthalate-*co*-ethylene furanoate) (rPETF) copolymers obtained from chemically recycled PEF and PET. The tensile strength and strain data for PET_Bottle_ are as reported by Niskanen *et al.*^34^

Sample name	DSC				TGA	Tensile test	
	*T*_g_ (°C)	*T*_m_ (°C)	Δ*H*_m_ (J g^−1^)	*X*_c_ (%)	*T*_d,5%_	Tensile strength (MPa)	Tensile strain (%)
PET_Bottle_	87	235	32.3	23.1	384	55.2 (ref. 34)	3.6 (ref. 34)
PEF_Reactor_	82	188	7.5	5.5	341	24.3 (±12.1)	1.44 (±0.30)
rPEF	83	192	48.6	35.5	371	25.9 (±4.4)	2.15 (±0.42)
rPETF 25–75	81	—	—	—	382	42.9 (±5.2)	4.17 (±0.85)
rPETF 50–50	74	—	—	—	368	10.6 (±3.4)	0.80 (±0.12)
rPETF 75–25	77	186	8.1	5.8	400	43.2 (±3.9)	3.43 (±0.83)
rPET	83	225	33.4	23.9	384	46.3 (±5.7)	20.8 (±2.83)

The degradation temperatures (*T*_d_) of the copolymers increase with increasing TPA content from 371 °C to 400 °C. Both Konstantopoulou *et al.* and Sousa *et al.* report that the degradation of PEF (*T*_d_ 398 °C) starts at lower temperatures compared to PET (*T*_d_ 450 °C).^60,79^ The DEG content in the polymers also affects the degradation temperature. The *T*_d_ of PEF_Reactor_ is 30 °C lower than that of rPEF, the *T*_d_ of rPETF 50–50 is 14 to 32 °C lower than the *T*_d_ of the other two copolymers, and the *T*_d_ of rPET is observed at 384 °C, 50 °C lower than what has been reported for PET, however, we measured a similar *T*_d_ 384 °C for PET_Bottle_, see Table 3.^60,79^ The main degradation mechanism for both PEF and PET is heterolytic scission of the chains combined with homolytic (radical) degradation at higher temperatures.^60^ The TGA thermograms are presented in the SI, Fig. S12.

**Fig. 5:**
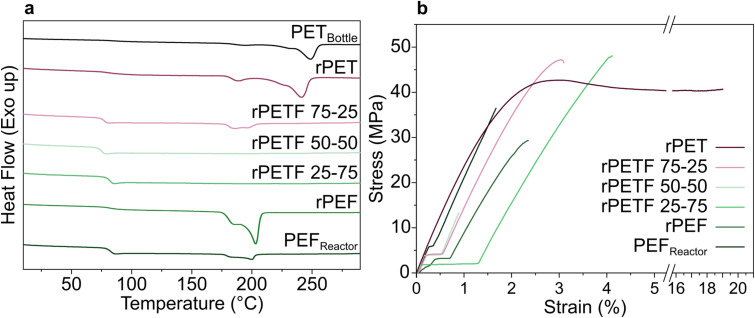
(a) DSC thermograms (2nd heating) and (b) tensile stress/strain curves of poly(ethylene furanoate) (PEF); and re-poly(ethylene furanoate) (rPEF) and re-poly(ethylene terephthalate-*co*-ethylene furanoate) (rPETF) copolymers obtained from chemically recycled PEF and PET.

Tensile strength curves are presented in Fig. 5b, and the average tensile strength and strain of the materials in Table 3. PEF_Reactor_ and rPEF had similar tensile strength results to previously reported tensile strengths of PEF (29.8–40.7 MPa).^34^ The incorporation of TPA in the copolymers increased the tensile strength for both rPETF 25–75 and rPETF 75–25, 42.9 and 43.2 MPa, respectively. Further, the tensile strength of rPET is 46.3 MPa and the tensile strain is 20.8%. However, these polymers have higher molecular weights compared to PEF_Reactor_ and rPEF (*M*_w_ 50.9 and 63.5 kg mol^−1^*vs.* 94.1–113 kg mol^−1^), which contribute to the higher tensile strength and increased tensile strain of these polymers. In addition, rPEF and rPET have significantly higher degrees of crystallinity. For comparison, the tensile strength of PET is reported to be 55.2 MPa with 3.6% tensile strain.^34^ rPETF 50–50 had very low tensile strength and strain at break (10.6 MPa and 0.8%), compared to the other polymers. This could be due to the non-existent crystallinity of the 50–50 copolymers as was also observed by Konstantopoulou *et al.*^60^ PEF_Reactor_ has slightly lower (24.3 MPa) tensile strength than rPEF (25.9 MPa). The increase of DEG from 6.7% (rPEF) to 10.7% (PEF_Reactor_) had only a minor effect on the mechanical properties of the polymers. Strain-induced crystallization is observed as the plateau in the beginning of the tensile testing (Fig. 5b), which is a known phenomenon for polyesters such as PET.^80^ This indicates that crystallization of the polymers can be induced by strain, although the thermal crystallization of copolymers containing both TPA and FDCA is not favoured.

As such, the properties of the obtained copolymers are different from the virgin homopolymers, unless they only contain a small fraction (<5%) of either TPA in PEF or FDCA in PET, respectively. To overcome this limitation, the copolymerization process should be optimized (time and temperature) for each copolymer separately, with regards to obtain sufficient molecular weight and melt viscosity of the copolymers to yield copolymers with mechanical properties comparable to the virgin materials. The introduction of soft segments or monomers (DEG, butanediol, *etc.*) into the copolymers to make them more ductile should also be investigated. In addition, catalysts other than titanium isopropoxide should be investigated to reduce side reactions, such as discoloration and uncontrolled formation of DEG, during polymerization.

## 4. Conclusions

To address some of the concerns (incompatibility, material properties, quality *etc.*) of recycling of bio-based polymers as drop-in alternatives for fossil-based plastics, we compared the chemical recycling of fossil-based PET and bio-based PEF, followed by repolymerization of the obtained oligomers and monomers into homo- and copolymers. Chemical recycling of PET and PEF and the combining of the feeds can overcome some of the challenges with introducing drop-in alternatives. However, the properties of the polymers combined in the feed should be similar to start with, which is the case with PET and PEF.

The chemically recycled oligomers and monomers (BHEF and BHET) could be polymerized into high-molecular weight rPEF (*M*_w_ 50.9 kg mol^−1^) and rPET (113 kg mol^−1^). Copolymers containing 25, 50, or 75% FDCA in the feed yielded copolymers with 23, 48, and 77% FDCA in the obtained copolymers and *M*_w_ values of 94.1, 123.4, and 109.9 kg mol^−1^. For comparison, the *M*_w_ of commercial PET from a soda bottle was 55.8 kg mol^−1^. Due to the difference in the melting temperatures of PET (265 °C) and PEF (192 °C), the reaction conditions and temperature should be tailored based on the feed composition of BHET and BHEF. The PEF homopolymer and the copolymer with 75% TPA (rPETF 75–25) were found to be semi-crystalline polymers, whereas the other two copolymers (rPETF 25–75 and rPETF 50–50) with 25 and 50% TPA were amorphous. The copolymers did, however, show crystallization under strain, and the polymers showed tensile strengths similar to what has been reported before for PET and PEF.

To conclude, we have shown that the chemical recycling of PET and PEF followed by repolymerization can provide the means to recycle both PET and PEF in the same recycling feed. However, it is vital that the FDCA content in the final polymer is rather low, or *vice versa*, the TPA content in PEF is low. In this case, the properties are similar to PEF or PET homopolymers. The copolymers with compositions of 75 : 25, 50 : 50, or 25 : 75 all turned out to be rather brittle. One way to improve the properties of the copolyesters would be to increase the molecular weight further to match the intrinsic viscosity of the virgin polymers and improve the ductility of the polymers by introducing soft segments. Hence, collecting PEF and PET waste in the same recycling feed and chemically recycling them together seems feasible only if the mass fraction of either polymer is rather low in the feed, or the recyclate is complemented with virgin polymer, which would still reduce our dependency on fossil-based monomers.

## Author contributions

Lauri Välinen: conceptualization, formal analysis, investigation, methodology, writing – original draft. Hossein Baniasadi: formal analysis, methodology. Jukka Niskanen: conceptualization, funding acquisition, supervision, writing – review and editing.

## Conflicts of interest

There are no conflicts to declare.

## Supplementary Material

RA-016-D6RA02625G-s001

## Data Availability

The data supporting this article have been included as part of the supplementary information (SI). Supplementary information: ^1^H-NMR spectra, size exclusion chromatography results with PMMA calibration, DSC curves with 120 °C isothermal step, TGA curves and reactor stirring torque graphs. See DOI: https://doi.org/10.1039/d6ra02625g.
