# Supplementary material for: Chemical recycling and repolymerization of poly(ethylene terephthalate), poly(ethylene furanoate), and their copolyesters
Source: RSC Adv. 2026 Jul 6. Online ahead of print. doi: 10.1039/d6ra02625g (PMC13335686; doi:10.1039/d6ra02625g)
Supplement: RA-OLF-D6RA02625G-s001 [file RA-OLF-D6RA02625G-s001.pdf]

Supplementary Information (SI) for RSC Advances.
This journal is © The Royal Society of Chemistry 2026

Chemical recycling and repolymerization of poly(ethylene terephthalate), poly(ethylene furanoate), and their copolyesters

Lauri Välinen,[a] Hossein Baniasadi[a] and Jukka Niskanen[a*]

[a] *Polymer Synthesis Technology, School of Chemical Engineering, Aalto University, Kemistintie 1, 02150, Espoo, Finland*

Supporting information

Full nuclear magnetic resonance spectra

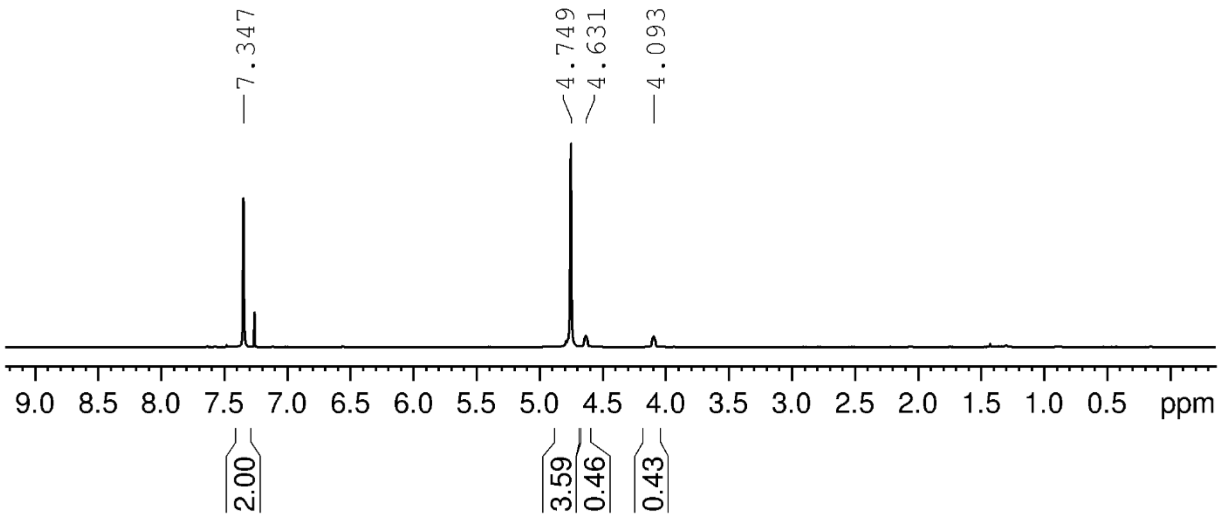

Figure S1. ^{1}H NMR spectra of $PEF_{Reactor}$

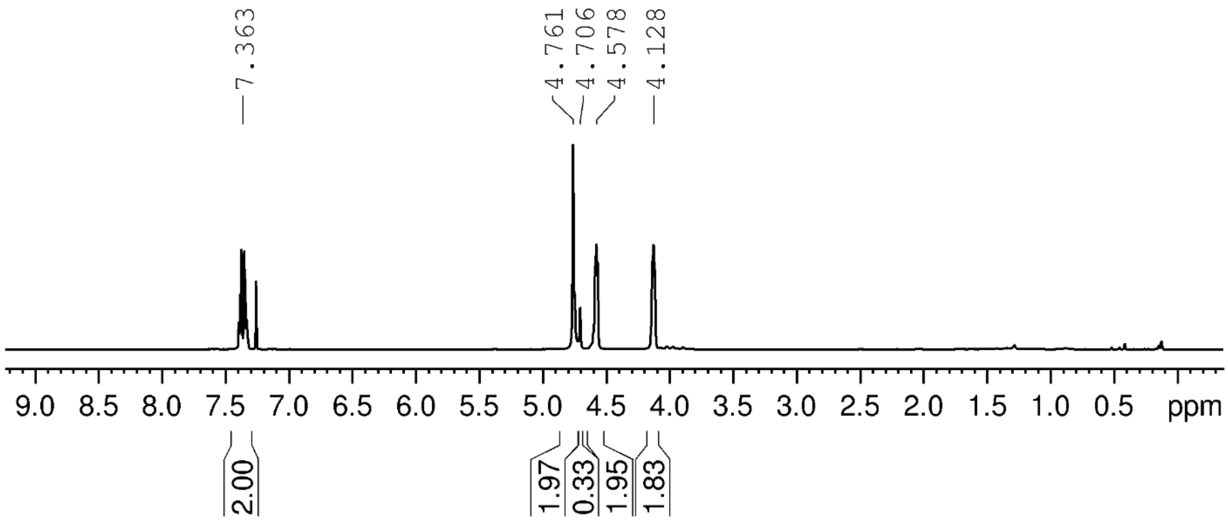

Figure S2. ^{1}H NMR spectra of BHEF

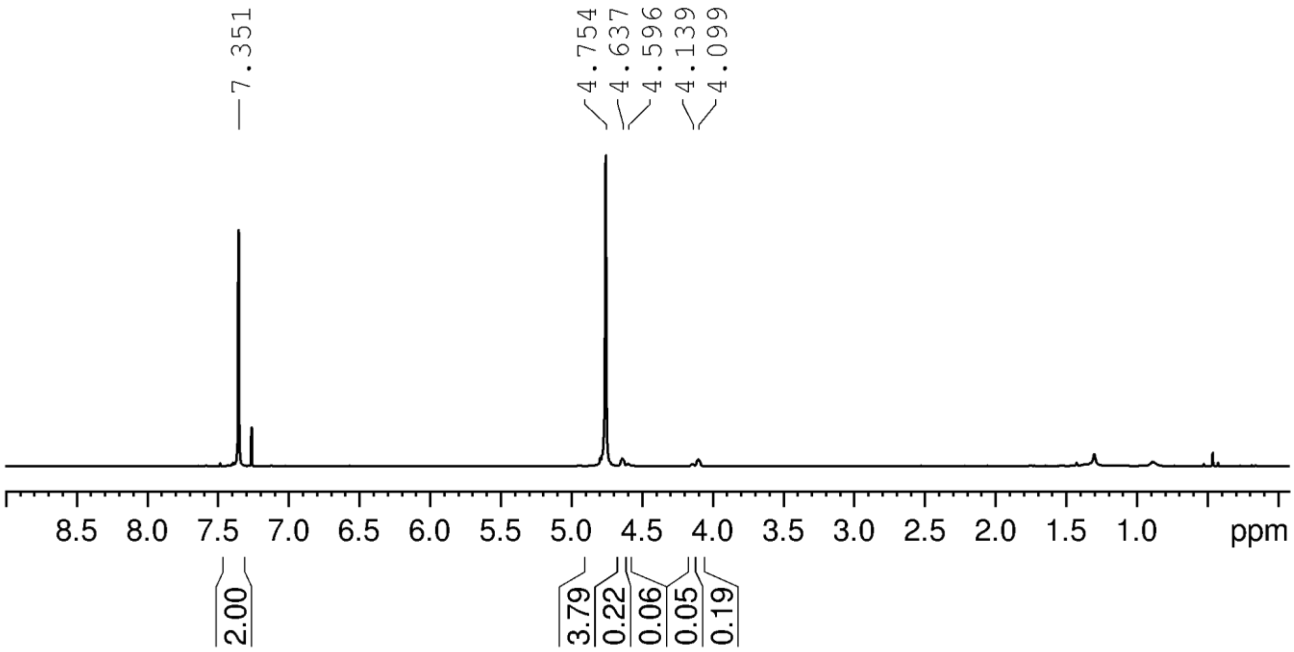

Figure S3. ^{1}H NMR spectra of PEF_{Flask}

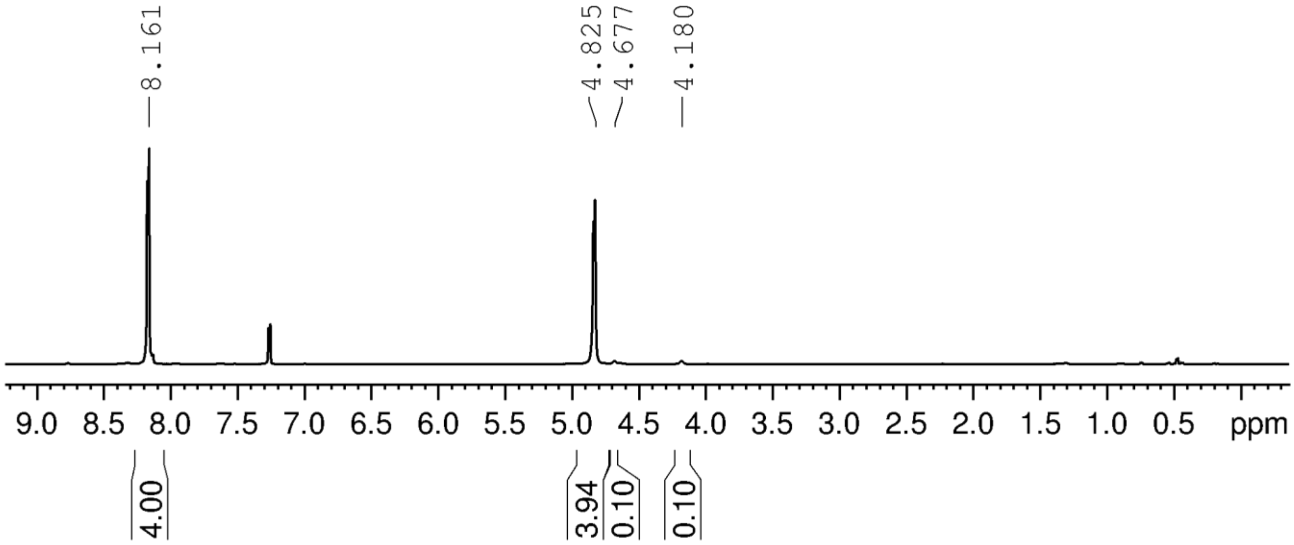

Figure S4. ^{1}H NMR spectra of PET

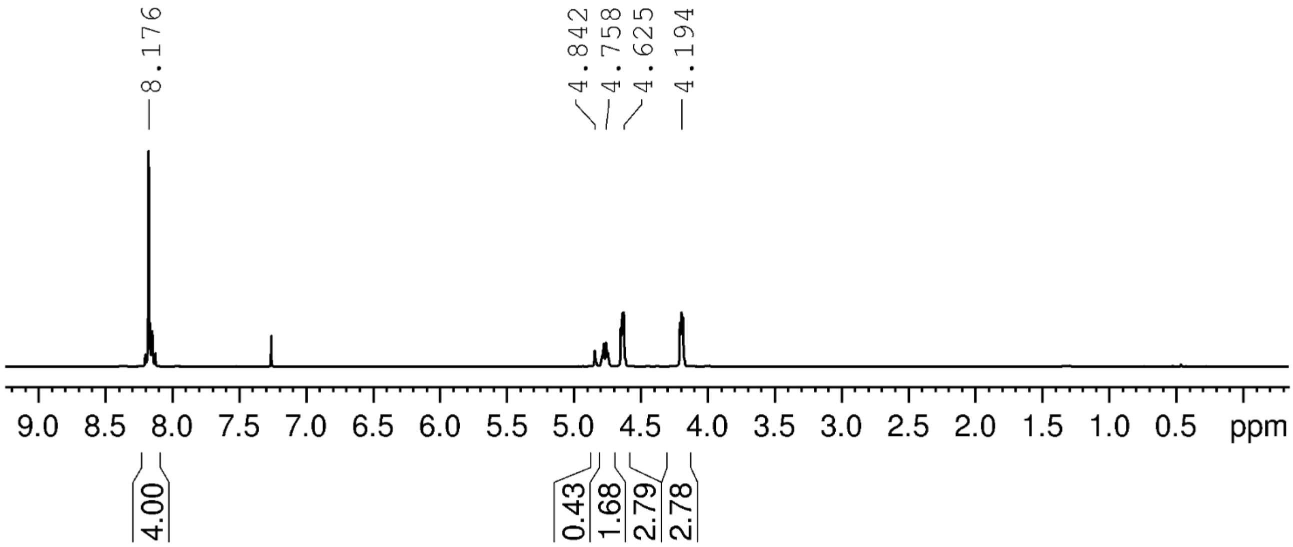

Figure S5. ^{1}H NMR spectra of BHET

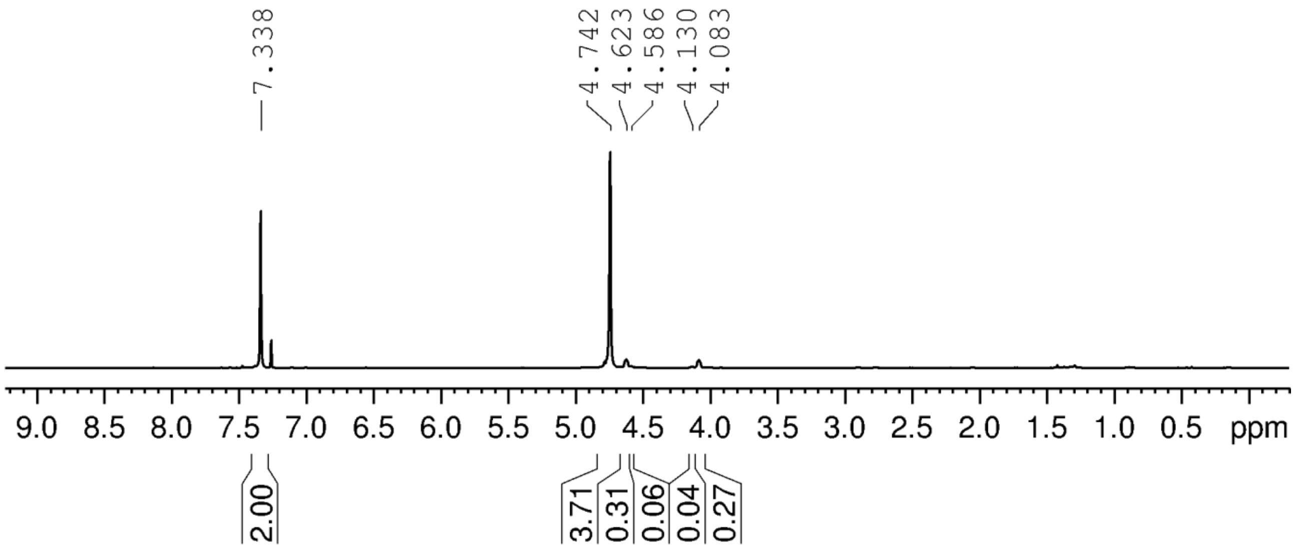

Figure S6. ^{1}H NMR spectra of rPEF

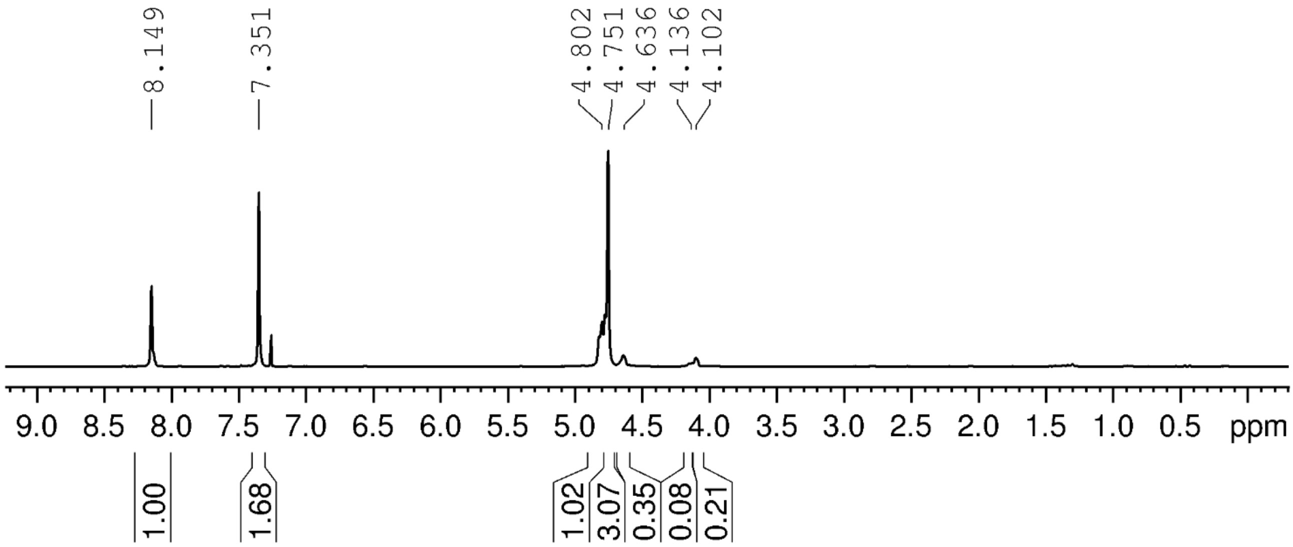

Figure S7. ^{1}H NMR spectra of rPETF$_{25\text{-}75}$

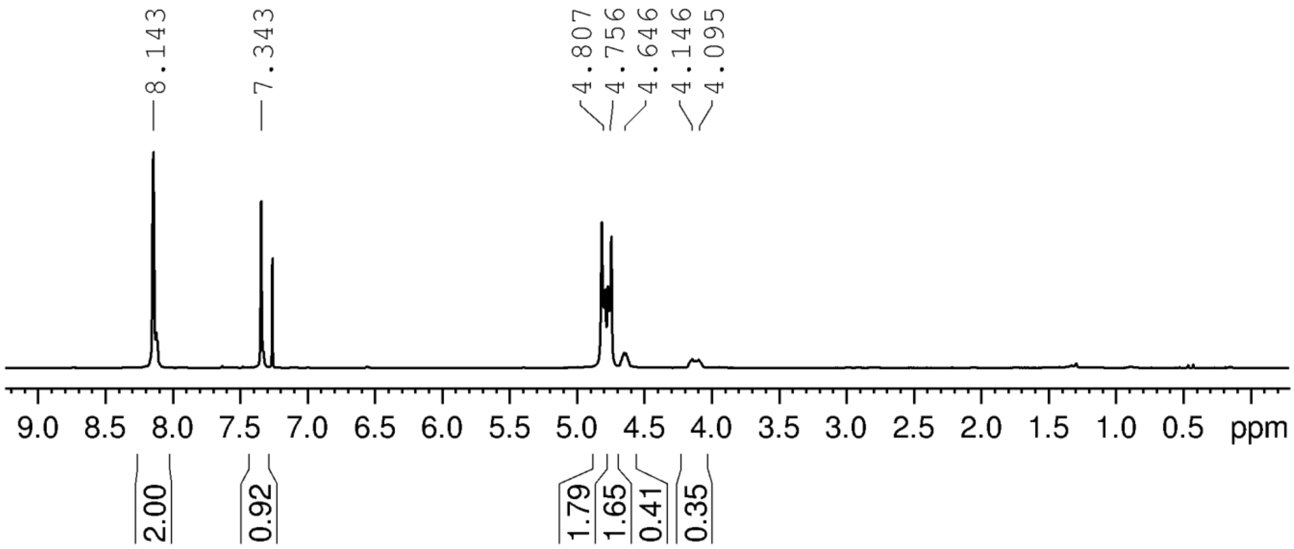

Figure S8. ^{1}H NMR spectra of rPETF$_{50\text{-}50}$

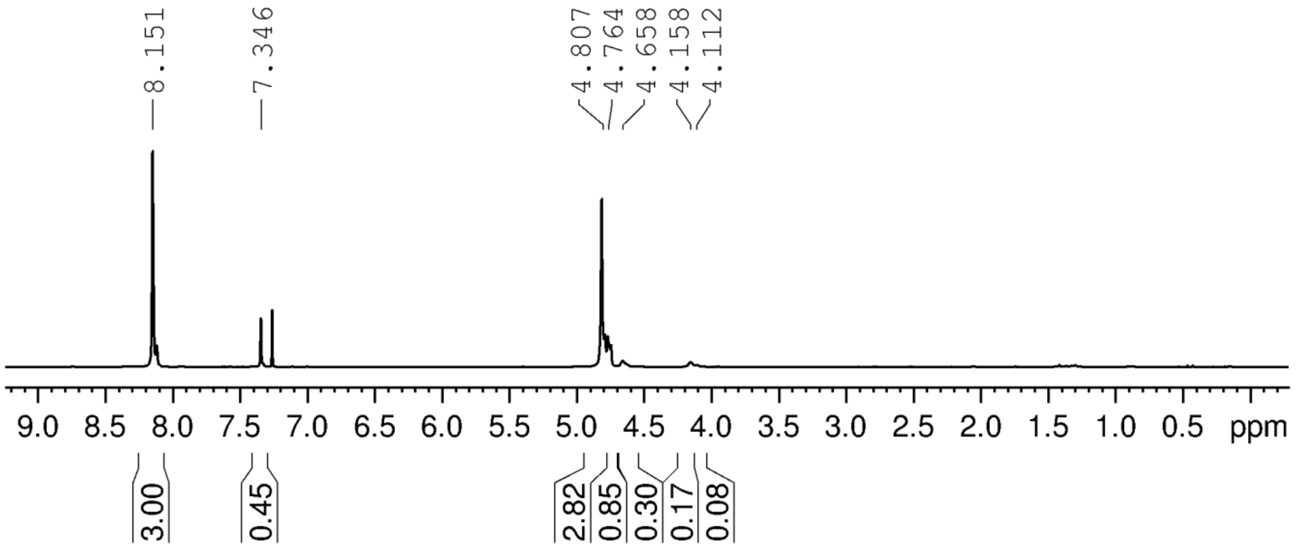

Figure S9. ^{1}H NMR spectra of rPETF75-25

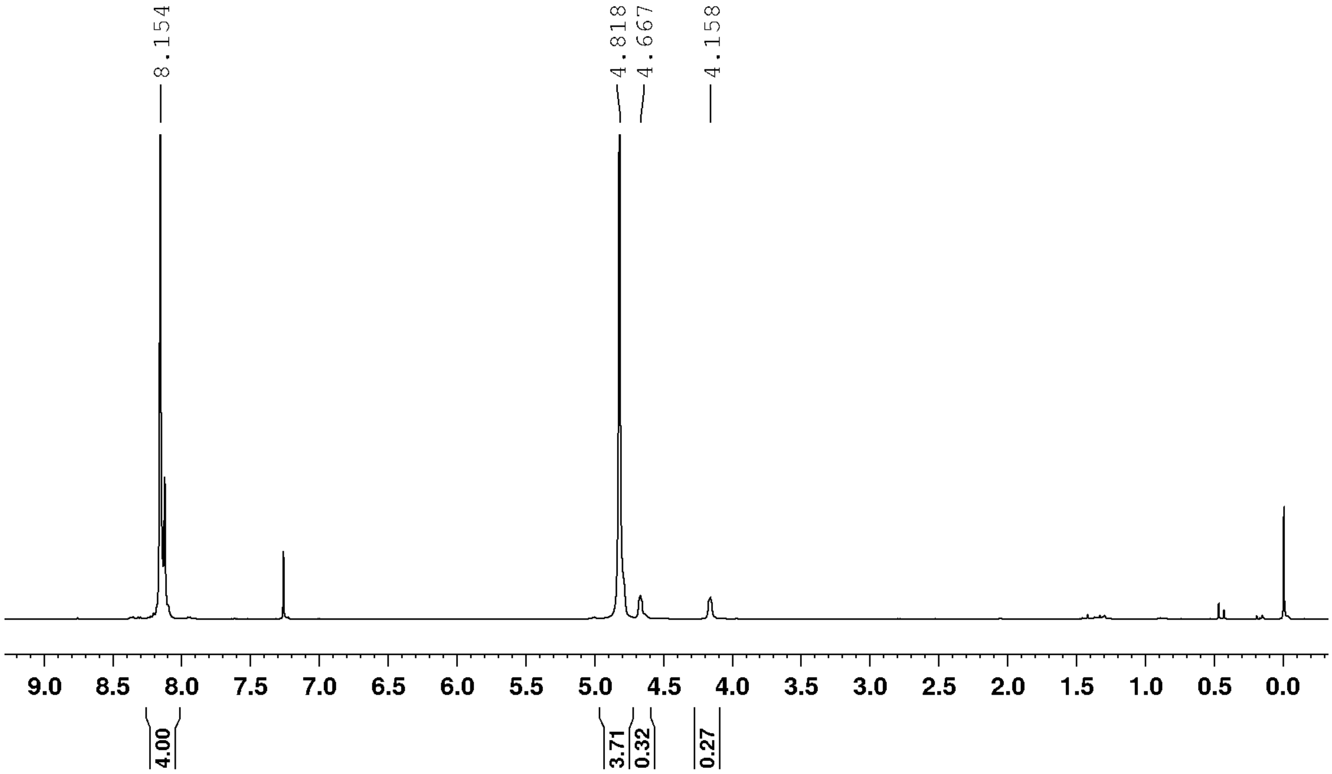

Figure S10. ^{1}H NMR spectra of rPET

Table S1 size exclusion chromatography results with PMMA calibration

SEC PMMA calibration			
Sample	Mn (kDa)	Mw (g/mol)	Đ
PET	18 800	47 600	2.53
PEF_{Flask}	19 200	43 600	2.28
$PEF_{Reactor}$	28 300	91 700	3.23
rPEF	25 600	111 700	4.37
rPETF25-75	28 300	154 500	5.47
rPETF50-50	23 400	123 000	5.27
rPETF75-15	21 000	104 100	4.95
rPET	24 100	74 000	3.07

Dynamic scanning calorimetry

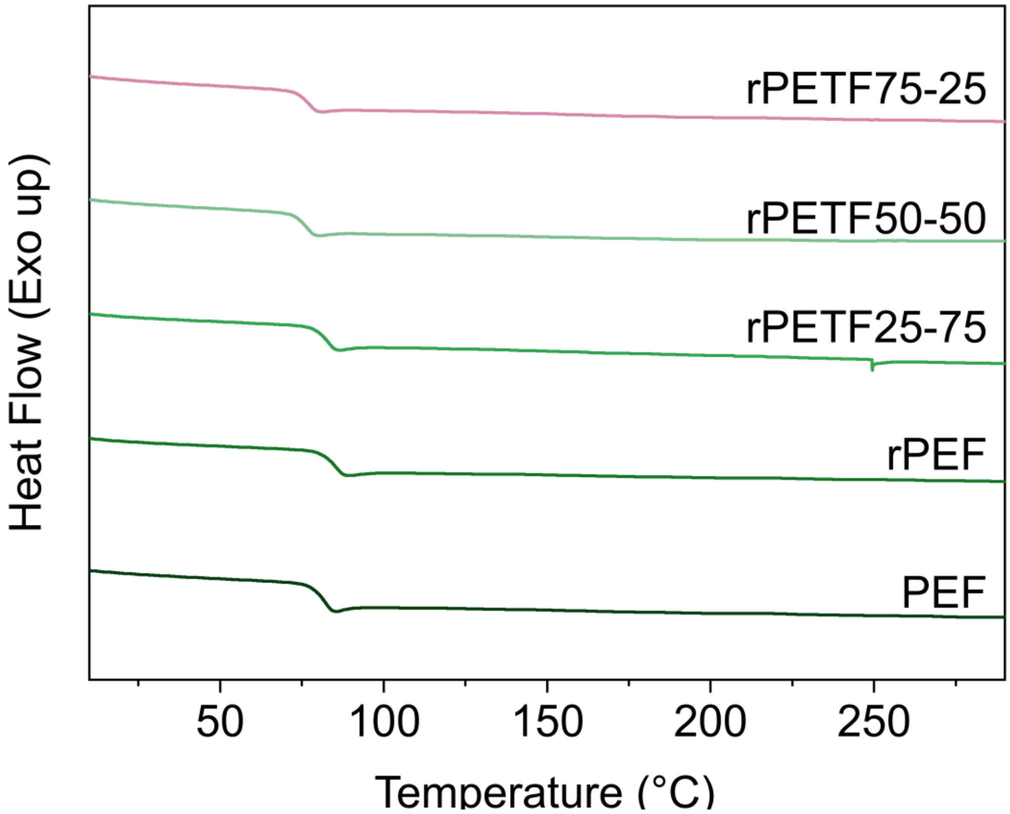

Figure S11. DSC With isothermal step at 120 °C

Thermal gravimetric analysis

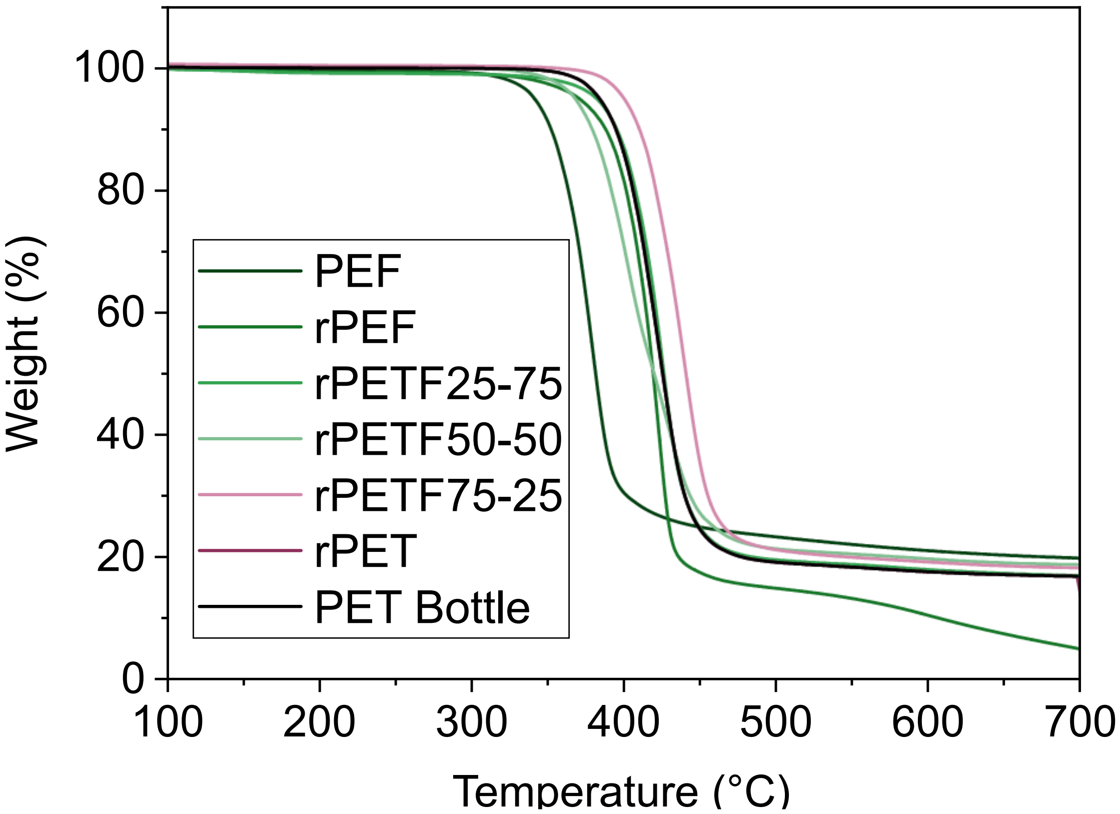

Figure S12. TGA

Reactor stirring torque

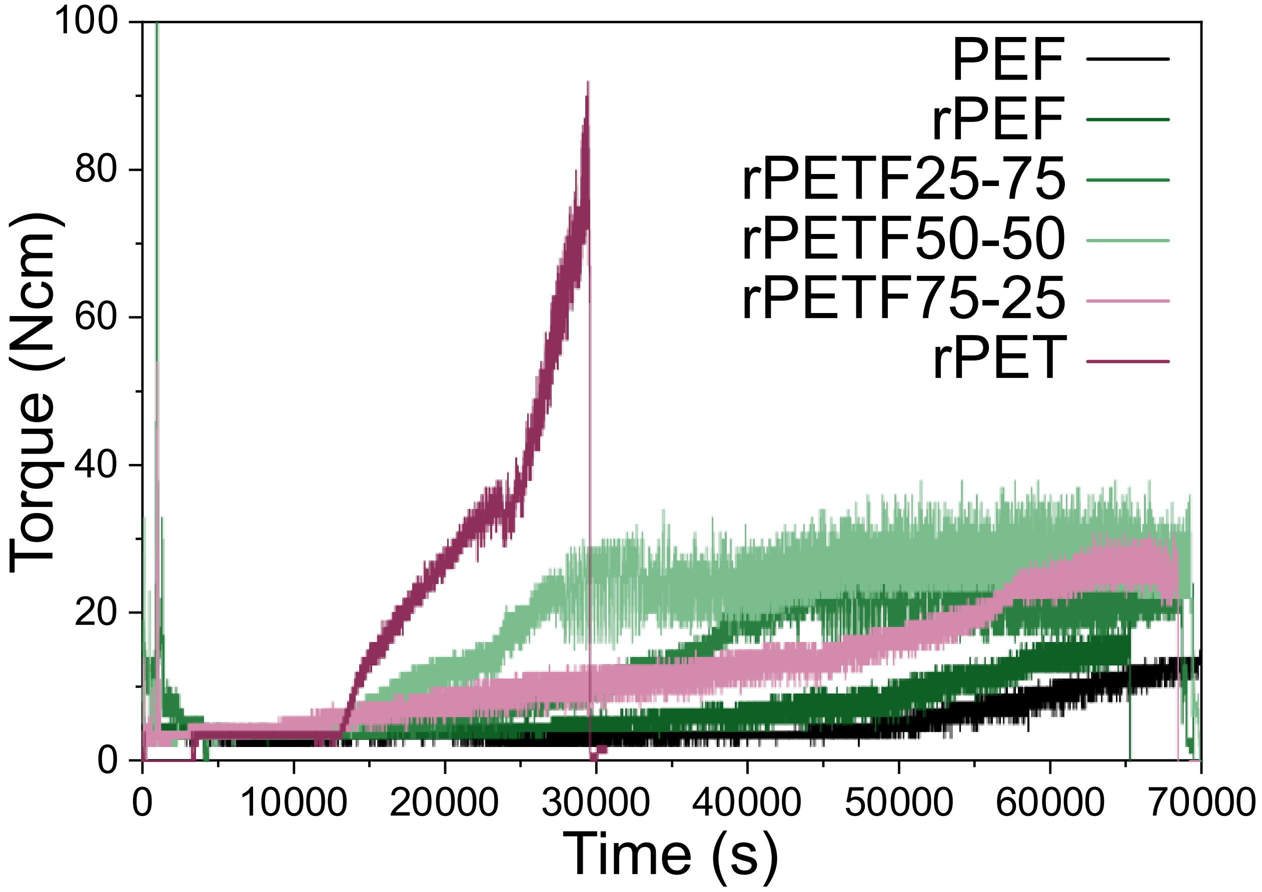

Figure S13. Reactor stirring torque
